# GenDiS database update with improved approach and features to recognize homologous sequences of protein domain superfamilies

**DOI:** 10.1093/database/baz042

**Published:** 2019-04-03

**Authors:** Meenakshi S Iyer, Kartik Bhargava, Murugavel Pavalam, Ramanathan Sowdhamini

**Affiliations:** 1 National Centre for Biological Sciences, Tata Institute of Fundamental Research (TIFR), Gandhi Krishi, Vignana Kendra Campus, Bellary Road, Bangalore, Karnataka, India; 2 Birla Institute of Technology and Science, Pilani, VidyaVihar Campus, Pilani, Rajasthan, India

## Abstract

Since proteins evolve by divergent evolution, proteins with distant homology to each other may or may not bear similar functions. Improved computational approaches are required to recognize distant homologues that are functionally similar. One of the methods of assigning function to sequences is to use profiles derived from sequences of known structure. We describe an update of the Genomic Distribution of protein structural domain Superfamilies (GenDiS) database, namely GenDiS+, which provides a projection of SCOP superfamily members on the sequence space (NR database, NCBI). The sequences are validated using structure-based sequence alignment profiles and domain and full-length sequence alignments. GenDiS+ is a `tour de force’ for detecting homologues within around 160 000 taxonomic identifiers, starting from nearly 11 000 domains of known structure. Features, like full-sequence alignment and phylogeny, domain sequence alignment and phylogeny, list of associated structural and sequence domains with strength of interactions, links to databases like Pfam, UniProt and ModBase and list of sequences with a PDB structure, are provided.

## Introduction

The number of protein sequences is estimated to be three orders of magnitude higher than the number of structures currently available in PDB (1). The number of folds being limited (2) and homologous proteins having similar structures and protein sequences can be classified into the existing folds using sequence profiles. Profiles derived from Position-Specific Scoring Matrix (PSSM) (3), Hidden Markov Model (HMM) (4) and Markov random field-based (5) methods are found to be very sensitive in accurately classifying sequences into structural folds or families. Several approaches exist for classifying a sequence into structural folds. These methods rely on different approaches like threading, contact-prediction, homology-based methods and support vector-based methods.

Databases like Superfamily (6) and Gene3D (7) provide information on homologues of known structure from 2487 complete genomes and genomes in UniProtKB (8), respectively. The NR database, NCBI, has sequence information for 564 020 taxonomic identifiers including unclassified organisms. Genomic Distribution of protein structural domain Superfamilies (GenDiS) database provides information about homologous sequences, starting from domains of known structure and belong to the same superfamily. The connections are performed at the sequence level along with the taxonomic distribution. The users can also compare different genomes for a particular domain superfamily and analyse the other co-existing domains. The users can also obtain information about the domain architecture (DA) of the homologues and the associated superfamily of the co-existing domains. GenDiS has been used for deriving profiles from superfamily hits for sequence searches or for an estimate on the number of proteins for a family in a genome (9–13), and the methodology has been adapted in other studies (14). In this paper, we describe an update of the database with additional features. The new release of GenDiS+ records 18 million homologous domains, around 23 million co-existing SCOP domains, around 38 million Pfam domains from 1965 SCOP superfamilies and over 27% of approximately 65 million scanned sequences (from around 1.6 million organisms). The results have also been presented for homologues, starting from domains represented from 1195 folds and 7 classes in SCOP 1.75.

## Materials and methods

**Figure 1:**
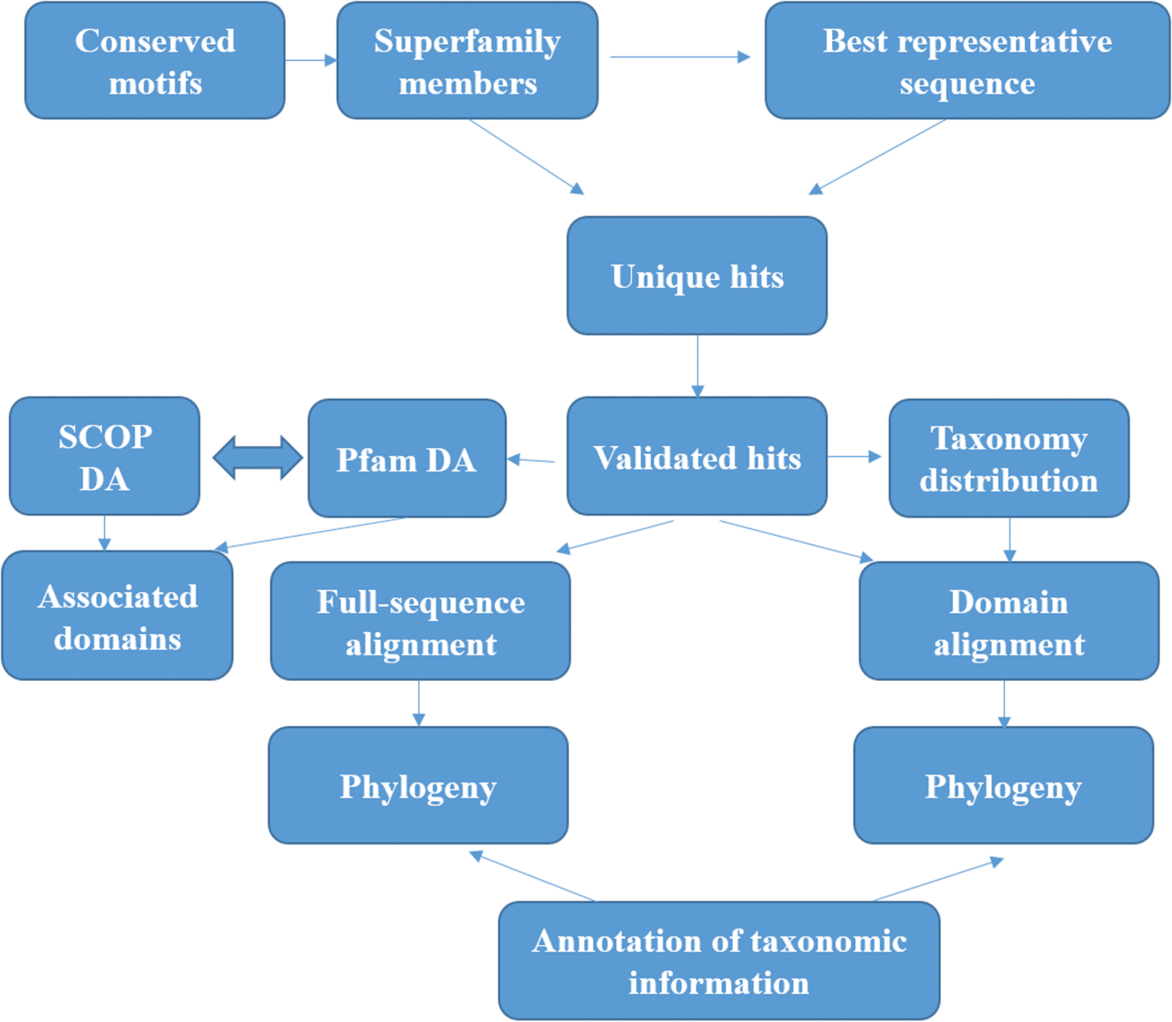
**Workflow followed for GenDiS+.** The sequence searches were carried out using BRS-MP approach for multi-membered superfamilies, as described in the Materials and Methods section. The hits were validated using a stringent validation using structure-based sequence alignments of superfamily members. From the validated hits, features like DA computation and phylogeny, domain and full-length alignment and phylogeny and conserved motifs were analysed and have been made available in the database.

The methods for sequence search and validation have been described in our previous paper (15) and in Figure 1. However, we describe them briefly here.

### Searching for homologues of structural members

Two approaches were used for identifying homologues, the first being multi-query (MQ) approach with all PASS2.4 (16, 17) superfamily members as queries. The second approach was using multiple patterns per query for the single-membered and two-membered PASS2.4 superfamilies and a best-representative sequence-multi pattern (BRS-MP) approach for multi-membered superfamilies. CSI-BLAST (18) was used using MQ approach, while PHI-BLAST (19) was used for MP approach with an *E*-value and inclusion threshold of 10^−3^ for 20 iterations. The sequence search was carried out against NR database, NCBI (May 2015).

### Validation and analysis

In the PASS2 database, structural alignments are created for the superfamily members (as organized in SCOP) but with <40% sequence identity with each other. In this manner, the alignment is not skewed by sequences with high sequence identities. In some superfamilies, structurally deviant members were removed, and structural alignments were re-constructed to obtain good-quality alignments (17). HMMs were created from the PASS2.4 structure-based sequence alignments of domain superfamilies (Superfamily/SF-HMM) and individual sequences (Single query/SQ-HMM) using HMMER suite (20). Each of the hits was assessed if they are true positives, using the HMMs and program HMMSCAN from HMMER. HMM matches with an independent *E*-value (i *E*-value) of 10^−3^ and model coverage of 0.7 were considered. HMM overlaps of up to 25 residues were allowed, beyond which the match with lower i *E*-value was considered. Finally, the sequences with at least a single HMM match of the same superfamily as the query were assigned as true positive hits. Discontinuous domains were identified using continuity of HMM coordinates and discontinuity of sequence coordinates (explained in Supplementary File).

The presence of co-existing domains of known structure was recognized through HMM library derived from SCOP database. Henceforth, we refer to such connections, and DA is referred as SCOP-DA. Likewise, the presence of co-existing domains, which are mainly recorded in sequence domain databases, such as PFAM, was recognized using search against a library of PFAM (v 28) HMMs. Henceforth, this result is referred as Pfam-DA. DA of homologues was compared, and homologues were clustered using an in-house tool called alignment-free DA similarity search (ADASS) algorithm (21). The strings of domains between sequences are used to score the extent of dissimilarity amongst sequences employed to create phylogeny. Taxonomy assignments were carried out using NCBI Taxonomy database (22). Domain regions were extracted from sequences and aligned using Clustal Omega (23), and full-length sequences were aligned using MAFFT (24). Trees were computed for the alignments using Clustal Omega and PartTree (25) program, respectively.

### Database setup

The database was constructed using HTML, CSS, Bootstrap and json-like collections derived from MongoDB using Robo3T. MongoDB collections were created for the details like taxonomic distribution, SCOP and Pfam DA, SCOP fold and class mapping, taxa and DA mapping and PASS2.4 HMM details for each. Search options were implemented using python scripts using Jinja as the interface between python and HTML. The web interface can be accessed from different browsers (Firefox, Chrome and Microsoft Edge) and operating systems (Windows, Linux and MacOS) and has been aesthetically designed for both desktop and laptop screens.

## Results

### Search and browse GenDiS+

A quick search can be carried out against the database, using keywords like the six-digit SCOP superfamily code, SCOP superfamily name, Pfam family name, NCBI accession identifiers and SCOP and Pfam DAs. A fuzzy search has been implemented for superfamily and family names. The database can be browsed using three different SCOP hierarchies—class, fold and superfamily. The taxonomy browsing option projects trees to display NCBI taxonomic ranks like kingdom and superphyla, and the tree branches are connected to links displaying the lower taxonomic ranks by hyperlinks. The database can also be searched using NCBI taxa names like species name, family, phylum or kingdom names using the `Browse by taxonomy’ tab.

**Figure 2:**
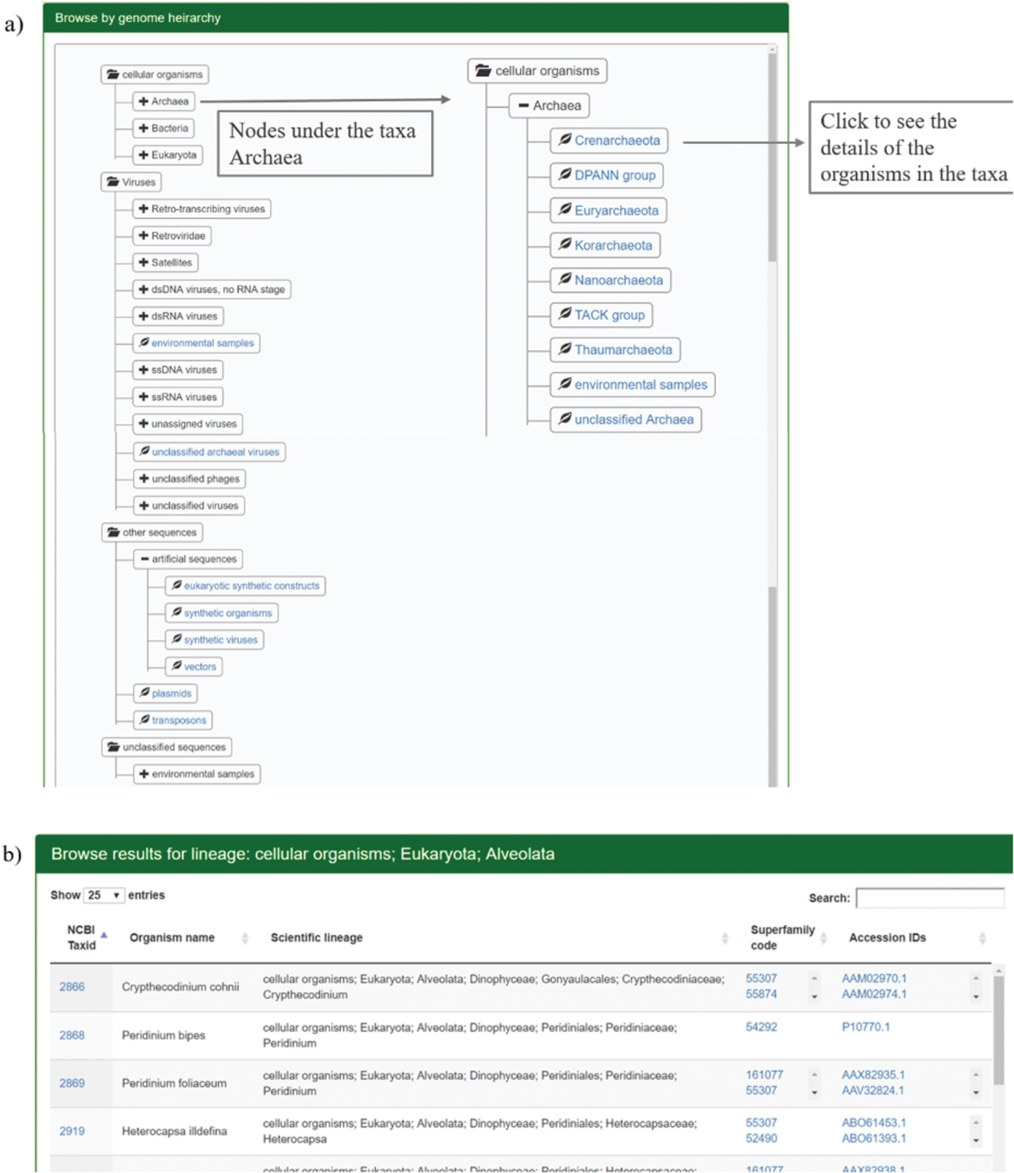
**Results for browsing by taxonomy.** (a) The taxonomy page lists the superphyla, phyla and family under different categories like cellular organisms, viruses, unclassified sequences and artificial sequences. The users can click on the parent nodes to expand the contents. Clicking on the child nodes with a leaf sign leads to a page listing all the species in the node with the complete hierarchy, accession ids and SCOP superfamilies of homologues. Links have been provided to NCBI taxonomy database on clicking the taxids and GenBank/Refseq page on clicking protein accession ids. Results for the expanded branch of the kingdom `Archaea’ has been shown. (b) Users can also browse the database using names for taxa, phyla, family etc. An example for search result of the keyword `Alveolata’ is shown.

The users can browse the database through SCOP or taxonomic hierarchy and at the species level. The user can browse through SCOP classes, folds or superfamilies using the `Browse by SCOP hierarchy’ option. On clicking the class option, a page is displayed that lists the folds and superfamilies under each SCOP class (Figure 2a). All the folds in SCOP (v1.75) are displayed in the webpage on folds, with the respective classes and superfamilies (Figure 2b). Links are provided for the corresponding structural class and folds in SCOP and superfamily information in GenDiS+.

The distribution of homologues in each species can be analysed for different hierarchies like cellular organisms, viruses, unclassified organisms and artificial sequences. Each of these hierarchies is divided into different superphyla, phyla and families. The page has been rendered using Anytree module from python, styled by Bootstrap CSS. By clicking the sub-taxa levels, the user can browse the different organisms under each sub-taxon. The taxid, species name and taxonomic hierarchy are displayed along with the structural superfamilies that occur in the proteome and their accession ids. Links are provided for the taxid, accession ids from NCBI and superfamily to the GenDiS+ superfamily page. Sort buttons are provided for each column to sort the entries numerically or alphabetically. The coverage of different taxonomic hierarchies has been provided in Table 1.

### Information and downloads

**Figure 3:**
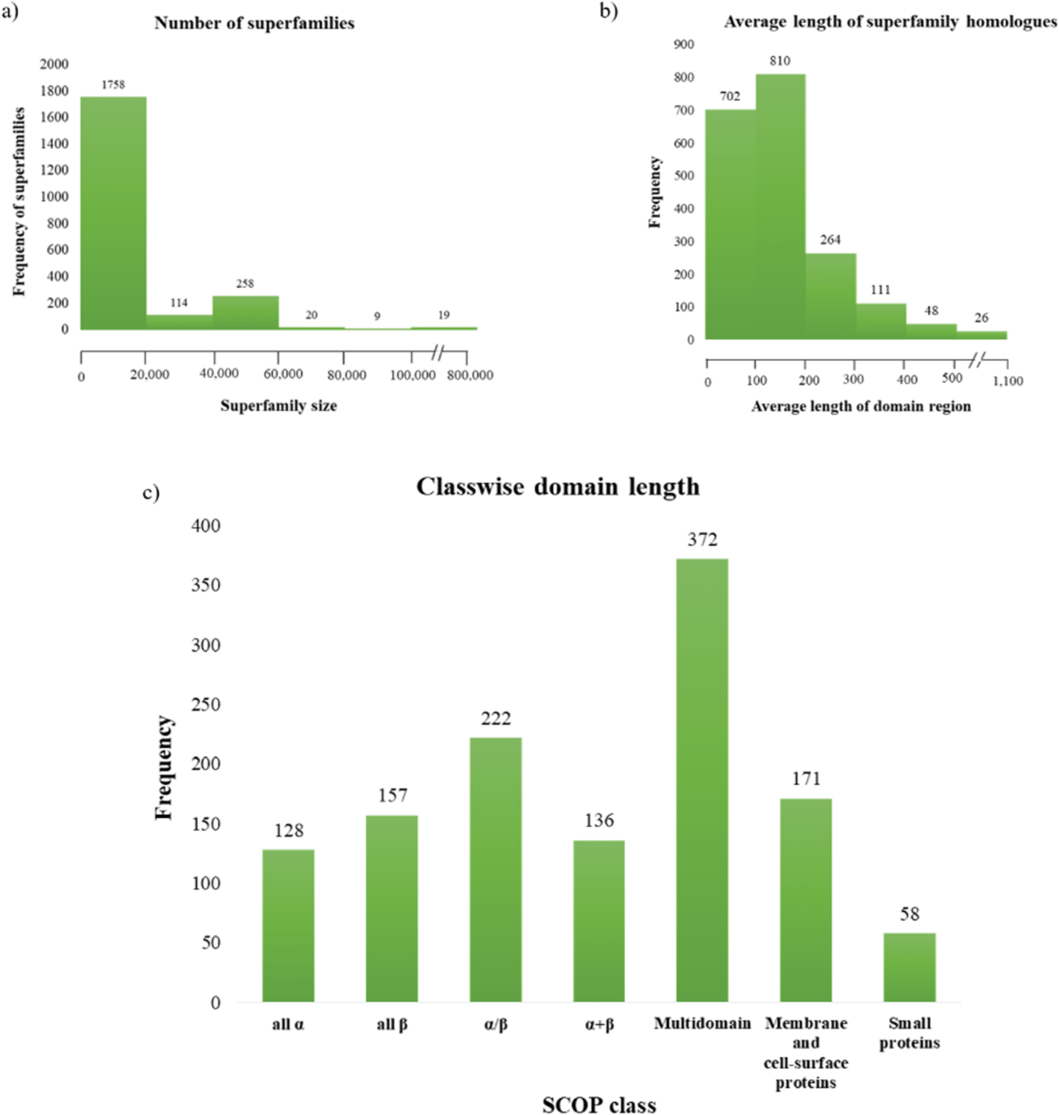
**Superfamily statistics in GenDiS+. (**a) The distribution of homologues across superfamilies. A majority of the superfamilies (89.65%) have up to 20 000 homologues with only 19 (~1%) superfamilies having over 100 000 members. (b) Distribution of domain length across superfamilies. (c) Most of the superfamily homologues have an average length up to 200 residues, with only homologues of multi-domain superfamily class having a domain length above 500 residues.

The main `Downloads’ page can be used to retrieve homologous sequences annotated using our pipeline, PASS2 structure-based sequence alignment HMM (SF-HMM) and single PASS2 member HMM (SQ-HMM). This includes the combined SF-HMM and SQ-HMM libraries, full-length and extracted domain regions for all the superfamilies. The full-length sequences represent the fraction of sequence space with at least a single domain, which is homologous to a superfamily and can be used as a database for sequence searches. The statistics page provides information about sequence coverage of different databases and superfamily member statistics (Tables 1 and 2 and Figure 3).

**Table 1:** Coverage of different databases

Database	Level	Total	Covered	% Coverage
Pfam	Families	16 230	9853	60.7
SCOP v1.75	Superfamilies	1962	1961	99.9
SCOPe v2.06	Superfamilies	2008	1961	97.7
NR, NCBI	Sequences	67 289 356	18 325 278	27.2
UniProt	Sequences	9 666 472	48 000 767	20.1
PDB	Protein structures	130 536	81 026	62.1
SwissProt	Sequences	557 275	298 699	53.6
Taxonomy database, NCBI	Organisms	164 890	67 377	40.9

The coverage of different sequence databases like Pfam, NR, UniProt and SwissProt and structural databases like PDB, SCOP and taxonomy database NCBI has been listed. The Pfam coverage of 60% is the highest reported so far.

**Table 2:** Coverage of different taxa in the NCBI taxonomy database

Taxa	Number of organisms in Taxonomy database	Number of organisms in GenDiS+	% Coverage
Archaea	2017	1999	99.1
Bacteria	37 639	36 901	98.0
Eukaryota	304 175	67 376	22.1
Fungi	18 562	6253	33.7
Viridiplantae	82 529	21 869	26.5
Metazoa	191 380	35 723	18.7
Other sequences	6569	1296	19.7
Unclassified organisms	235	137	58.3
Viruses	112 389	47 850	42.6
Total	488 685	156 363	32.0

The coverage of different kingdoms and superphyla in the taxonomy database, NCBI, has been described. Most of the archaeal and bacterial genomes have been covered, whereas lower coverage is seen for Eukaryotes especially, Metazoa and Viridiplantae.

A library of NCBI organisms with taxonomic identifiers, complete lineage and the superfamily domains identified (as taxid library) has also been provided for download. A library of the SCOP-DA and Pfam-DA, identified in the study, has been provided for download as SCOP-DA library and Pfam-DA library, respectively. List of superfamilies (specific to taxa such as archaea, bacteria, metazoa, viridiplantae, fungi and viruses) and superfamilies common to the three kingdoms of life are also available for download.

### Superfamily description page

**Figure 4:**
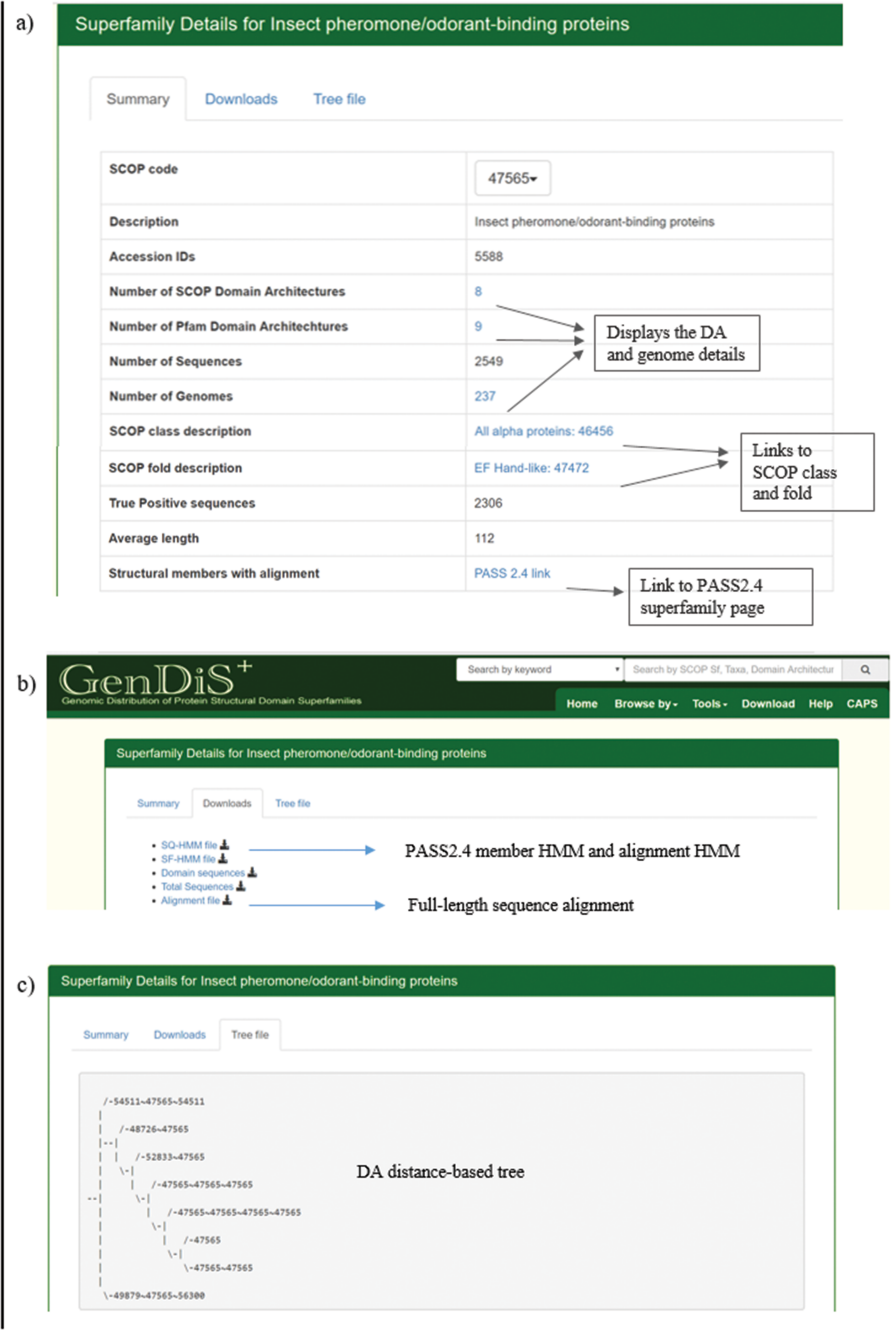
**Superfamily description page.** Details pertaining to each superfamily like (a) SCOP/Pfam DA, genomes covered, full length are available in the summary page; (b) validated sequences, domain sequence alignment and phylogeny are available for download in the `Downloads’ page; and (c) the DA-distance trees can be visualized in the `Tree file’ page.

The superfamily description page can be accessed by searching the database using the SCOP code or superfamily name and from the `Browse by Superfamily’ option. The `Summary’ tab displays details of the superfamily such as the SCOP code, description, SCOP class and fold, number of hits, organisms, SCOP-DA and Pfam-DA (Figure 4a). Links are provided for the NCBI taxids, SCOP-DA and Pfam-DA of superfamily members. The number of sequences, organisms in which the homologues are found, SCOP-DA and Pfam-DA are mentioned with colour codes for the quartiles in which the numbers stand with respect to all superfamilies. The taxonomic distribution, SCOP-DA and Pfam-DA are listed with the NCBI accession identifiers in the corresponding links. A list of the SCOP superfamilies and Pfam families, associated with each superfamily, is provided with the number and frequency of DAs in the `SCOP Domain’ and `Pfam Domain’ pages, respectively. From the `Downloads’ page, users can download the PASS2.4-based superfamily and single query HMMs (SF-HMM and SQ-HMM), alignment of the extracted domain regions after validation, full-sequence alignment of homologues and the sequence phylogeny (Figure 4b). The full-length sequence phylogeny is provided with a metadata file for annotation of organisms in the tree at the species and family level. The `Tree file’ tab displays the SCOP DA tree as derived from ADASS tool (Figure 4c). The `Members’ tab displays PASS2 superfamily members with links for the sequence and conserved motifs file.

The domain alignments and the HMMs of PASS2 superfamilies can be used for sequence searches or for validation of hits obtained from sequence searches carried out for specific superfamilies.

### Tools

To facilitate analysis of the sequence data, three different tools have been made available in GenDiS+ and have been described in detail in Supplementary File. These tools enable analysis of particular domain superfamilies of interest.

**Table 3:** The performance of different profiles for the superfamily Acyl-Carrier Protein (ACP)-like (family PP-binding) in identifying hits from different model organism proteomes

	*Homo sapiens*		*Escherichia coli*		*Arabidopsis thaliana*		*Caenorhabditis elegans*	
	TP	FP	TP	FP	TP	FP	TP	FP
Pfam	9	1	**3**	**0**	13	0	7	4
PASS2.5	5	0	2	0	10	0	6	1
GendiS+-Domain	8	0	2	0	13	0	6	0
GenDiS+-Full-length	7	5	4	2	17	6	7	5
GenDiS+-Trimmed	**9**	**0**	4	1	**13**	**0**	**7**	**3**
GenDiS+-Symfrac	**9**	**0**	**3**	**0**	**13**	**0**	**7**	**3**

The profile with the best performance has been highlighted in bold. The GenDiS+ profile of full-length alignments with 70% column information performs the best in all the cases.

TP, true positives; FP, false positives.

**Table 4:** The coverage of PDB entries in different databases for the superfamily SMAD MH1 domain by our method

Databases	Total members	Members covered
PASS2.4	1	1
SCOP 1.75	1	1
SCOPe (v2.06)	3	3

All the PDB entries listed in the below databases were retrieved in the sequence searches. An entry from mouse that was not covered by the following databases was also recovered indicating that we have been able to improve coverage at both sequence and structure space.

### Applications: case study of Smad protein MH1 superfamily

The superfamily SMAD MH1 (MAD homology 1) domain (SCOP code 56366) has SCOP entries from human and mouse. We found homologues for the superfamily in 297 proteomes from the phyla Cnidaria and Bilateria, thereby sampling superphyla that are not included in PDB such as Protostomia and Platyhelminthes.

The coverage of PDB entries from different databases by our method has been shown in Table 3. The two protein sequences covered in SCOP (v1.75) are from humans, whereas SCOPe (v2.07) has an entry from mouse (3KMP) #ref; the other entry that is 51% identical to the sequence from mouse has not been classified (3QSV) (Baburajendran *et al.*, unpublished results).

**Figure 5:**
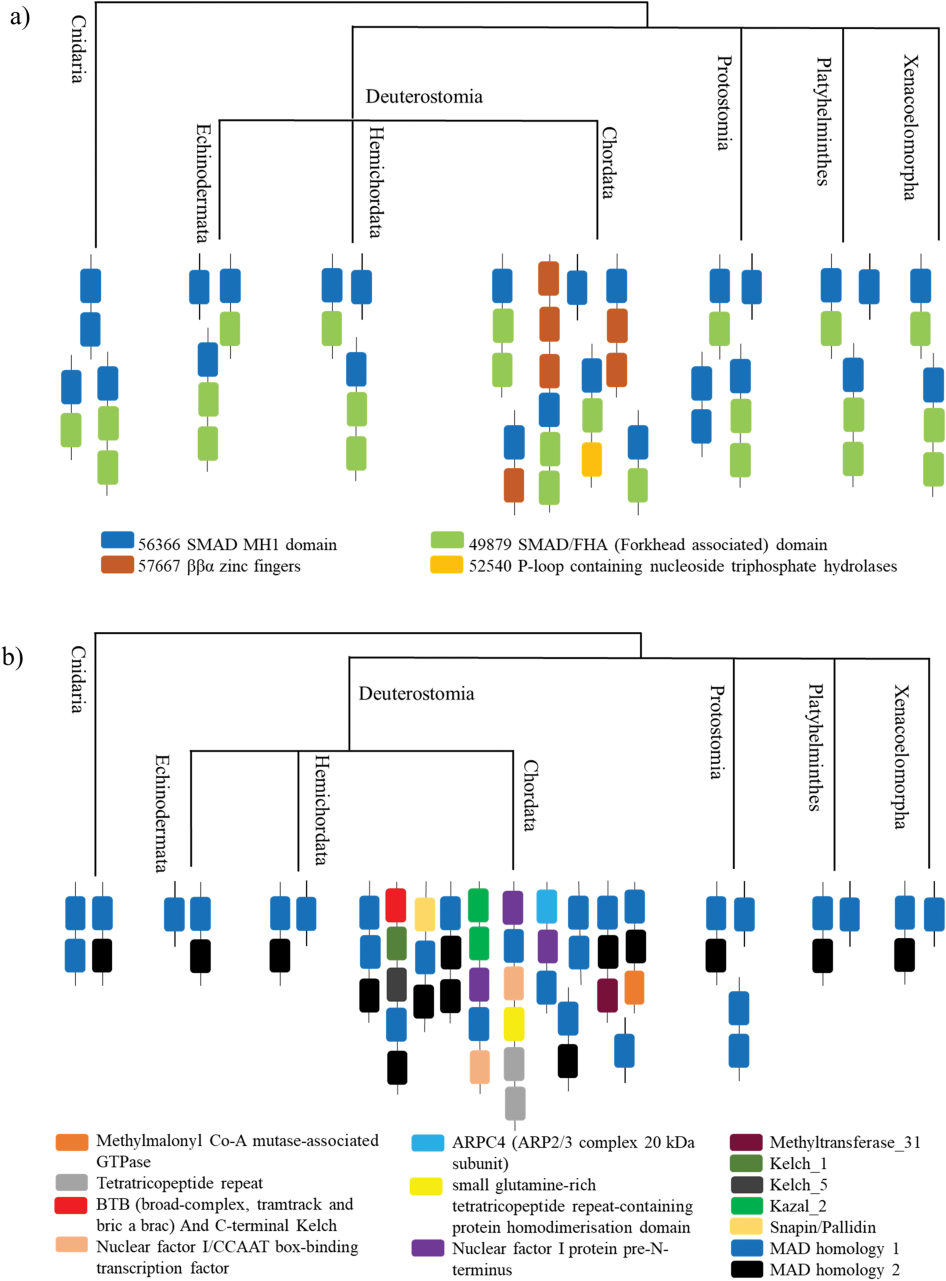
**A case study of Smad protein MH1 superfamily. (**a) The phylogenetic distribution of the SCOP-DA is represented, across different taxa in Cnidaria and Bilateria. As shown, the single-domain form and the combination with SMAD/FHA are prevalent in all orders while the other domain combinations occur only in certain taxa. (b) The phylogenetic representation of Pfam DA indicates that there is a correspondence between DA predictions by both methods in spite of using differently derived HMM libraries.

The most prevalent SCOP-DA is the associated DA with SMAD/FHA domain, followed by the single domain of SMAD MH1. Other less prevalent SCOP-DA contain double-domain form of SMAD MH1 domain, β-β-α zinc fingers and P-loop containing nucleotide triphosphate hydrolases. Similarly, the Pfam-DA consists of the single- and double-domain forms and combinations with domains like SMAD MH2 domain, Snapin/Pallidin, GTPase ArgK and others (Figure 5a and b).

All the organisms contain the associated DA with SMAD/FHA domain and the single-domain form, whereas the double-domain form occurs in Cnidaria and Protostomia. Chordata contains all the other DA, which has the maximum domain architectural diversity.

### Applications of GenDiS+ database in sequence searches

Profiles were derived from GenDiS+ domain sequence alignments, GenDiS+ full-sequence alignments with and without trimming of gaps and using information from columns ,which are present in at least 70% of the sequences for the acyl carrier protein-like superfamily. SCOP profiles were derived from PASS2.5 (26), and Pfam family profiles were also used for the comparison. The performance of the above profiles was compared with respect to the identification of annotated hits in the proteomes from different model organisms (UniProt Reference proteome). The GenDiS+ full-length profile (derived from alignment positions with at least 70% sequence conservation) performed as well as Pfam profiles with respect to number of hits and true positives (Table 4).

## Discussion

Sequence annotation is the most important step in genomic, metagenomic and transcriptomic studies. The number of structures available in PDB and SwissProt is far less compared to the number of sequences in the sequence database, which poses a hurdle in the functional and structural annotation. We report the update of a database, GenDiS+, to bridge the gap between the sequence space and structural space using sensitive computational searches and profiles of structural entries of members of a superfamily. Our database is unique (in comparison to other databases like PDB, SCOP and PASS2.4) in providing additional information like sequence alignments, conserved motifs for homologues of a superfamily, DA for the identified homologues and taxonomic details. It is also different from databases like UniProt, Pfam and Superfamily in providing details for nearly 41% of organisms in NCBI Taxonomy database, rather than RefSeq proteomes or complete proteomes. Different details are provided for SCOP superfamilies, and files can be analysed and are available for download.

Using our approach, ~58% of sequences from unclassified and uncultured organisms and 30% of sequences from extrachromosomal origin like plasmids and transposons have been annotated. Information of the conserved motifs extracted from the alignment of homologues of superfamily members and associated SCOP superfamily and Pfam family domains (identified from the DA of homologues) has been provided. The full-length sequence alignments, domain sequence alignments and phylogeny have been provided with the taxonomy metadata. GenDiS+ database provides sequence information for validated superfamily members with links to other databases like PDB, SCOP, PASS2.4, UniProt and ModBase, if available.

The sequence alignment of the members is also available, which includes domains of known structures to validate the superfamily assignment. Both DA and sequence alignments, along with taxonomic distribution, can be used to understand the evolution of the superfamily. To the best of our knowledge, no other database provides structural annotations for entire taxonomy database and provides correspondence between SCOP and Pfam database with a high coverage (60%) of Pfam families. However, sequences with a novel fold or superfamily from PDB identifiers not classified in SCOP will be missed out in the tool. There are ~40% families in Pfam that have not been assigned a domain superfamily of known structure from our analysis. These families are good targets for structural genomics initiative, which will help in bridging the gap between the sequence and structural space.

## Supplementary Material

gendis_db_manu_supplemantary_file_revised_submit_baz042

gendis_revised_ST1_baz042

## Notes

**Database URL:** http://caps.ncbs.res.in/gendis+/

## References

[1] Lee J., Freddolino P.L. and Zhang Y. (2017) In: Ridgen DJ (ed)*.* *From Protein Structure to Function with Bioinformatics*. Springer, Dordrecht, Netherlands, pp. 3–35. doi:10.1007/978-94-024-1069-3_1.

[2] Chothia C. (1992) Proteins. One thousand families for the molecular biologist. *Nature*, 357, 543–544. 1608464 10.1038/357543a0

[3] Altschul S.F., Madden T.L., Schäffer A. et al. (1997) Gapped BLAST and PSI-BLAST: a new generation of protein database search programs. *Nucleic Acids Res.*, 25, 3389–3402. 9254694 10.1093/nar/25.17.3389 PMC146917

[4] Anand B., Gowri V.S. and Srinivasan N. (2005) Use of multiple profiles corresponding to a sequence alignment enables effective detection of remote homologues. *Bioinformatics*, 21, 2821–2826. 15817691 10.1093/bioinformatics/bti432

[5] Ma J., Wang S., Wang Z. et al. (2014) MRFalign: protein homology detection through alignment of Markov random fields. *PLoS Comput. Biol.*, 10, e1003500. 24675572 10.1371/journal.pcbi.1003500 PMC3967925

[6] de Lima Morais D.A., Fang H., Rackham O.J. et al. (2011) SUPERFAMILY 1.75 including a domain-centric gene ontology method. Nucleic Acids Res., 39, D427–D434. doi:10.1093/nar/gkq1130. 21062816 PMC3013712

[7] Buchan D.W., Shepherd A.J., Lee D. et al. (2002) Gene3D: structural assignment for whole genes and genomes using the CATH domain structure database. *Genome Res.*, 12, 503–514. 11875040 10.1101/gr.213802 PMC155287

[8] Apweiler R., Bairoch A., Wu C.H. et al. (2004) UniProt: the Universal Protein knowledgebase. *Nucleic Acids Res.*, 32, D115–D119. 14681372 10.1093/nar/gkh131 PMC308865

[9] Pugalenthi G., Tang K., Suganthan P.N. et al. (2007) A machine learning approach for the identification of odorant binding proteins from sequence-derived properties. *BMC Bioinformatics*, 8, 351. 17880712 10.1186/1471-2105-8-351 PMC2216042

[10] Sandhya S., Pankaj B., Govind M.K. et al. (2008) CUSP: an algorithm to distinguish structurally conserved and unconserved regions in protein domain alignments and its application in the study of large length variations. *BMC Struct. Biol.*, 8, 28. 18513436 10.1186/1472-6807-8-28 PMC2423364

[11] Tang K., Lin M., Minku F.L. et al. (2009) Selective negative correlation learning approach to incremental learning.*Neurocomputing*, 72, 2796–2805.

[12] Shah P.K., Tripathi L.P., Jensen L.J. et al. (2008) Enhanced function annotations for *Drosophila* serine proteases: a case study for systematic annotation of multi-member gene families. *Gene*, 407, 199–215. 17996400 10.1016/j.gene.2007.10.012

[13] Gai Y., Qiu L., Wang L. et al. (2009) A clip domain serine protease (cSP) from the Chinese mitten crab *Eriocheir sinensis*: cDNA characterization and mRNA expression. *Fish Shellfish Immunol.*, 27, 670–677. 19699801 10.1016/j.fsi.2009.08.005

[14] Bhaduri A. and Sowdhamini R. (2005) Genome-wide survey of prokaryotic O-protein phosphatases. *J. Mol. Biol.*, 352, 736–752. 16095610 10.1016/j.jmb.2005.07.004

[15] Iyer M.S., Joshi A.G. and Sowdhamini R. (2018) Genome-wide survey of remote homologues for protein domain superfamilies of known structure reveals unequal distribution across structural classes. *Mol. Omics*, 14, 266–280. doi:10.1039/c8mo00008e. 29971307

[16] Sowdhamini R., Burke D.F., Huang J.F. et al. (1998) CAMPASS: a database of structurally aligned protein superfamilies. *Structure*, 6, 1087–1094. 9753697 10.1016/s0969-2126(98)00110-5

[17] Gandhimathi A., Nair A.G. and Sowdhamini R. (2012) PASS2 version 4: an update to the database of structure-based sequence alignments of structural domain superfamilies. *Nucleic Acids Res.*, 40**,** D531–D534 (2012). 22123743 10.1093/nar/gkr1096 PMC3245109

[18] Biegert A. and Sö Ding J. (2009) Sequence context-specific profiles for homology searching. *PNAS.*, 106**,** 3770–3775. 19234132 10.1073/pnas.0810767106 PMC2645910

[19] Zhang Z., Schäffer A.A., Miller W. et al. (1998) Protein sequence similarity searches using patterns as seeds. *Nucleic Acids Res.*, 26, 3986–3990. 9705509 10.1093/nar/26.17.3986 PMC147803

[20] Finn R.D., Clements J. and Eddy S.R. (2011) HMMER web server: interactive sequence similarity searching. *Nucleic Acids Res.*, 39, W29–W37. 21593126 10.1093/nar/gkr367 PMC3125773

[21] Syamaladevi D.P., Joshi A. and Sowdhamini R. (2013) An alignment-free domain architecture similarity search (ADASS) algorithm for inferring homology between multi-domain proteins. *Bioinformation*, 9, 491–499. 23861564 10.6026/97320630009491 PMC3705623

[22] Federhen S. (2012) The NCBI Taxonomy database. *Nucleic Acids Res.*, 40, D136–D143. 22139910 10.1093/nar/gkr1178 PMC3245000

[23] Sievers F. and Higgins D.G. (2014) Clustal Omega, accurate alignment of very large numbers of sequences. *Methods Mol. Biol.*, 1079, 105–116. 24170397 10.1007/978-1-62703-646-7_6

[24] Katoh K., Misawa K., Kuma K. et al. (2002) MAFFT: a novel method for rapid multiple sequence alignment based on fast Fourier transform. *Nucleic Acids Res.*, 30, 3059–3066. 12136088 10.1093/nar/gkf436 PMC135756

[25] Katoh K. and Toh H. (2007) PartTree: an algorithm to build an approximate tree from a large number of unaligned sequences. *Bioinformatics*, 23, 372–374. 17118958 10.1093/bioinformatics/btl592

[26] Gandhimathi A., Ghosh P., Hariharaputran S. et al. (2016) PASS2 database for the structure-based sequence alignment of distantly related SCOP domain superfamilies: update to version 5 and added features. *Nucleic Acids Res.*, 44, D410–D414. 26553811 10.1093/nar/gkv1205 PMC4702857

