# Supplementary material for: GenDiS database update with improved approach and features to recognize homologous sequences of protein domain superfamilies
Source: Database (Oxford). 2019 Apr 3;2019:baz042. doi: 10.1093/database/baz042 (PMC6446967; doi:10.1093/database/baz042)
Supplement: gendis_db_manu_supplemantary_file_revised_submit_baz042 [file gendis_db_manu_supplemantary_file_revised_submit_baz042.docx]

**GenDiS database update with improved approach and features to recognise homologous sequences of protein domain superfamilies**

**Meenakshi S. Iyer^1^, Kartik Bhargava^1,2^, Murugavel Pavalam^1^ and Ramanathan Sowdhamini^1,^***

To facilitate analysis of the sequence data, three different tools have been made available in GenDiS+.

1. **Domain architecture prediction**

The user can provide a sequence in the input box and select an option for DA of SCOP or Pfam domain predictions. SCOP and Pfam DA prediction is carried out using HMMSCAN with the same libraries against this query sequence and options as outlined in the Methods section. Results of an example run has been shown in **Supplementary Figure 1a**.

1. **Align structural domains from a superfamily in two genomes**

The user has to select a superfamily from the dropdown menu and two genomes in the corresponding boxes. The homologous domains from both genomes are extracted and aligned using CLUSTALW^26^. This will help the user to study the conservation of residues within closely related or distantly related species of the superfamily. For example, in the alignment on **Supplementary Figure 1b**, there are five conserved cysteine residues.

1. **Align a given sequence with domain from a genome**

After identifying the domains of known structure (SCOP-DA) in a given sequence, the user can infer conservation of residues compared to sequences in any genome which contains the structural superfamily. The user provides the sequence in the sequence box, selects the superfamily and the genome from the drop-down menu (**Supplementary Figure 1c**). The given sequence will be aligned with the domain regions using CLUSTALW (Larkin et al. 2007).

The results of the tools can also be downloaded in a text format from the download link.

**
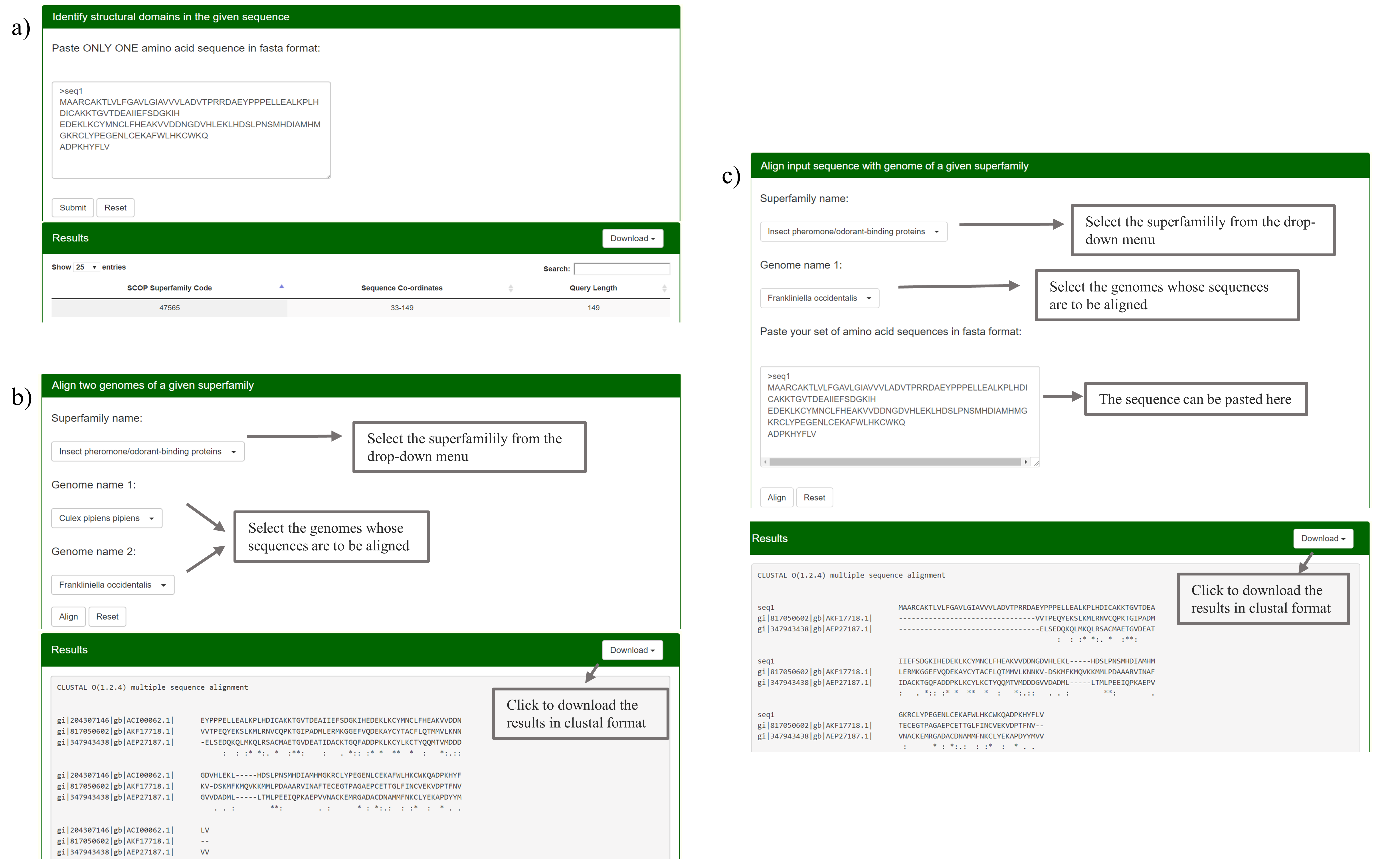
**

**Supplementary Figure 1: An overview of the tools and results**

a) The submitted sequence consists of a single domain of Insect pheromone/odorant-binding proteins superfamily (SCOP code: 47565). b) Domains belonging the superfamily Insect pheromone/odorant-binding proteins from the mosquito *Culex pipiens* and the thrip *Frankliniella occidentalis* were selected for sample runs. The alignment shows the six conserved cysteine residues from the typical OBP class. c) The sequence provided by the user has been aligned with the selected sequences from the superfamily 47565 and *F. occidentalis*.

**Identifying and assigning domains**

HMM coverage filter (mcf) of 0.7 and independent E-value (i E-value) of 0.001 was considered for assigning domains. In some cases, breaks were observed in HMM matches. This was due to sequence divergence in some regions or the presence of sequentially discontinuous domains. The former matches were identified by the envelope co-ordinates or HMM co-ordinates separated by up to 25 residues. The latter cases resulted due to insertion of secondary structural elements or a domain within a domain and were identified by the envelope and HMM co-ordinates being separated by more than 25 residues. The discontinuous HMM matches were considered, if the combined mcf was greater than 0.7 and the individual i E-values were less than 0.001. In case of more than one HMM match for the same region of a sequence, precedence was given to HMM match with the lowest i E-value.

**Benchmarking of the predictions**

The PDB hits from twelve superfamilies were analysed to check if they belong to the same superfamily using SCOPe and from the protein descriptions, in case the hits were not classified in SCOPe. Few entries classified in SCOP were missed for some of the superfamilies, but no false positives were identified. This indicates that the criteria used were stringent enough to avoid false positives. The results have been provided in **Supplementary Table 1**.
