# Supplementary material for: GenDiS database update with improved approach and features to recognize homologous sequences of protein domain superfamilies
Source: Database (Oxford). 2019 Apr 3;2019:baz042. doi: 10.1093/database/baz042 (PMC6446967; doi:10.1093/database/baz042)
Supplement: gendis_revised_ST1_baz042 [file gendis_revised_st1_baz042.docx]

**Supplementary Table 1**: The coverage of structural entries in GenDiS+ and other structural databases has been indicated. We have also been able to identify entries not classified in SCOP, indicating good coverage at both structural and sequence space.

| **SCOP super family code** | **Superfamily description** | **Number of PDB IDs identified in the sequence searches** | **% coverage of PDB IDs in SCOPe (v2.06)** | **Number of PDB entries not in SCOPe (v2.06)** |
| --- | --- | --- | --- | --- |
| 47336 | ACP-like | 70 | 76.51 | 8 |
| 47565 | Insect Pheromone-OBPs | 89 | 98.57 | 20 |
| 48345 | A virus capsid protein alpha helical domain | 9 | 100 | 4 |
| 50203 | Bacterial Enterotoxins | 198 | 95.94 | 9 |
| 51069 | Carbonic anhydrase | 496 | 74.32 | 7 |
| 51101 | Mannose-binding lectins | 76 | 91.30 | 34 |
| 51351 | Triosephosphate isomerase | 106 | 57.54 | 3 |
| 51679 | Bacterial-luciferase like | 20 | 86.36 | 1 |
| 51971 | Nucleotide-binding domain | 63 | 79.41 | 36 |
| 55031 | Bacterial exopeptidase dimerisation | 29 | 100 | 17 |
| 55239 | RuBisCo-small subunit | 44 | 97.78 | 0 |
| 55307 | Tubulin-CTD-like | 116 | 84.34 | 46 |
